# Characterization of CC-531 as a Rat Model of Colorectal Liver Metastases

**DOI:** 10.1371/journal.pone.0155334

**Published:** 2016-05-12

**Authors:** Sarah Beth White, Daniele Procissi, Jeane Chen, Venkateswara Rao Gogineni, Patrick Tyler, Yihe Yang, Reed A. Omary, Andrew C. Larson

**Affiliations:** 1 Department of Radiology, Division of Vascular & Interventional Radiology, Medical College of Wisconsin, Milwaukee, WI, United States of America; 2 Department of Radiology, Northwestern University, Chicago, IL, United States of America; 3 Department of Chemical and Biological Engineering, Northwestern University, Evanston, IL, United States of America; 4 Department of Radiology, Vanderbilt University, Nashville, TN, United States of America; 5 Department of Biomedical Engineering, Northwestern University, Evanston, IL, United States of America; 6 Robert H. Lurie Comprehensive Cancer Center, Northwestern University, Chicago, IL, United States of America; AntiCancer Inc., UNITED STATES

## Abstract

**Purpose:**

Surgical resection of colorectal liver metastases is not achievable in more than 70% of the cases. Although the liver directed therapies have become a part of the stand of care, lack of a preclinical model impedes the assessment of toxicity and therapeutic benefits attributed several candidate drugs or treatment regimens that can be designed. In the present study we aim develop and characterize a rat colorectal liver metastasis model.

**Materials and Methods:**

Growth characteristics of CC-531 cells were determined in vitro followed by subcapsular liver implantation in syngeneic WAG/Rij rats. Tumor growth progression was followed over 3 weeks by ultrasound (US) and magnetic resonance imaging (MRI). Growth characteristics were also assessed by histopathology and immunohistochemistry in harvested tumor tissues.

**Results:**

The doubling time of CC-531 cells was found be under 24hrs and all the implanted rats grew tumors. US imaging showed hypoechoic masses and MRI showed contrast enhancement representing complex tumor microenvironments. Hematoxylin and Eosin staining confirmed tumor growth and uniform CD31 staining in tumor confirmed even vessel density.

**Conclusion:**

CC-531 can be used as a metastatic rat tumor colorectal liver metastases model with well-defined characteristics that can be readily followed by imaging whilst having a therapeutic window for interventions.

## Introduction

Cancer is the 2^nd^ leading cause of death in the US at present and the American Cancer Society estimates that it will account for 577,190 deaths this year.[1] Of those deaths, 51,690 (9%) will be due to colorectal cancer (CRC), which is the third most common cause of cancer related death in the United States. Surgical resection of the primary tumor is curative; however, 19% of patients present with synchronous metastasis and most commonly these metastatic foci are in the liver. In 50 to 60% of all patients with CRC, liver metastases will develop at some point in the course of the disease. [2] Of the patients with colorectal liver metastases, only 25% are eligible for curative hepatic resection due to the number, size and location of their hepatic metastases.[3] According to the National Comprehensive Cancer Network (NCCN) guidelines, the first line therapy for treatment of metastatic colorectal cancer is Folinic Acid (leucovorin), Fluorouracil (5-FU) and Oxaliplatin (FOLFOX), or Folinic Acid, Fluorouracil and Irinotecan (FOLFIRI), or Capecitabine and Oxalipatin (CapOX) with or without the addition of cetuximab or panitumumab. [4] These chemotherapeutic regimens are extremely difficult for patients to tolerate, with only 63% of patients able to get a full 12 cycles.[5] In order to decrease systemic side effects of these toxic drugs, intra-arterial drug delivery coupled with embolization has become a fast growing adjuvant to traditional systemic chemotherapy. Currently, many studies including Drug-Eluting Bead, Irinotecan (DEBIRI), selective internal radiation therapy (SIRT) using yttrium-90 resin microspheres to standard fluorouracil, leucovorin, and oxaliplatin (FOLFOX)–based chemotherapy (SIRFLOX trial) and Enhanced Peri-Operative Care for High-risk patients (EPOCH trial) are underway to understand the role of liver directed therapies in the palliative treatment of colorectal liver metastases. [6, 7] For example, in a prospective randomized controlled trial evaluating DEBIRI vs. FOLFIRI, patients in the DEBIRI arm showed increased median overall survival (OS), 22 months vs. 15 months in the FOLFIRI arm. [8] Though these results are impressive, this procedure can be poorly tolerated due to its side effects, which were reported to be up to 19% in one study.[9]

Though liver directed therapies, such as ^90^Y radioembolization and drug eluting bead irinotecan transarterial chemoembolization [8, 10–12] are currently part of the treatment algorithm for colorectal liver metastases, there is still much that needs to be studied. For example, there is little knowledge about when liver directed therapy should be folded into current standard of care. In addition, patients with colorectal liver metastases may undergo multiple lines of chemotherapy and the effect of intra-arterial therapies after chemotherapy is not well known. There is also little known about whether genetic variations, such as K-ras mutations, will have an effect on treatment efficacy. Therefore, a preclinical model of colorectal liver metastases is necessary to help better understand the toxicities, timing and efficacy of liver directed therapies. The purpose of this study is to characterize CC-531, a rodent model of colorectal liver metastases.

## Materials and Methods

Institutional Animal Care and Use Committee (IACUC) of the Medical College of Wisconsin, Milwaukee, WI and Northwestern University, Chicago, IL has approved all the rat experiments. This study was carried out in strict accordance with the recommendations in the Guide for Care and Use of Laboratory Animals of the National Institutes of Health.

### Cell culture

The CC-531 cell line (generously donated by the University of Pittsburgh) was obtained and cultured in Dulbecco’s Modified Eagle Medium (DMEM) (Life Technologies, Carlsbad, CA) and supplemented with 10% fetal bovine serum (Gemini Bio-Products, West Sacramento, CA) and 1–5% Penicillin-Streptomycin (Sigma-Aldrich, St. Louis, MO) in culture flasks at 37°C in a humidified atmosphere containing 5% CO_2_. Cells were tested and found to be free of viruses and mycoplasma (Charles River, Wilmington, MA).

To determine the in vitro doubling time, 1 x 10^5^ CC-531 cells were plated in a 6 well plate. At 24 hours, the cells were rinsed twice with sterile phosphate-buffered solution (PBS) (Life Technologies) and then trypsinized with 0.5 mL of 0.25% trypsin (Thermo Scientific, Elkton, MD) for 5 minutes. The reaction was stopped with the addition of DMEM culture medium and the cell suspensions were transferred to 1.5 mL tubes and centrifuged at 1250 RPM for 5 minutes. Trypan blue *staining was performed* and the cells were counted using the Countess Cell Counter (Life Technologies). Additionally, 2.5 x 10^5^ cells were placed in three 25 cm flasks and allowed to grow for 68 hours. The cells were counted, as above and the doubling time was determined.

For implantation, the cells were cultured and maintained, as described above. After centrifugation, the supernatant was aspirated and the cells were resuspended in a known amount of DMEM. Trypan blue staining was performed and the cells were counted. The suspension was adjusted to ensure a density of 5 x 10^6^ cells in 0.25 mL of DMEM.

### Cellular inoculation

**Fig 1:**
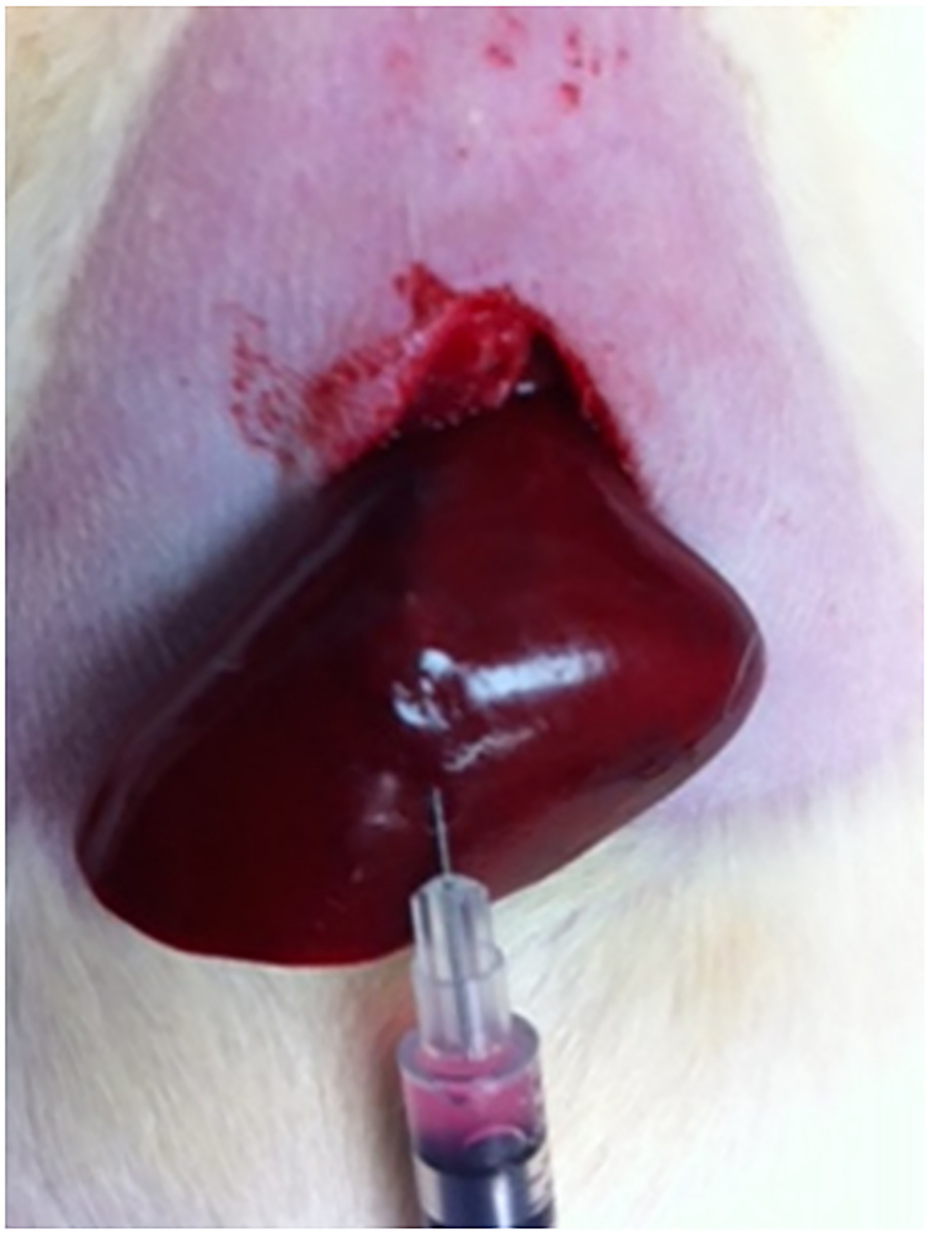
Tumor inoculation. Using a 29 G needle, 5*106 CC-531 cells in a cellular suspension were injected into the anterior subcapsular left hepatic lobe.

Because the CC-531 is syngeneic with the WAG/Rij rats, eight immunocompetent WAG/RijCmcr rats weighing 250-300g were used. Of note, the WAG/RijCmcr strain of rats (previously called WAG/RijMcw, WAG/Rij Y and WAG/Rij) are a biologically clean breeding colony that are not commercially available in the US, but bred locally [13–16]. The rats had a controlled climate and light cycles (in order to maintain their circadian rhythms), and all had free access to a standard laboratory diet and water. For the inoculation procedure, anesthesia was obtained using 2–3% Isoflorane. A 3 cm midline incision was made and both the left and right hepatic lobes were mobilized. The CC-531 cells in a cellular suspension with a density of 5 x 10^6^ are injected via a 29 gauge needle into the subcapsular portion of the previously specified hepatic lobes (Fig 1). When the needle was withdrawn, blood stop (PRN Pharmacal, Pensacola, FL) was used on the puncture site to achieve hemostasis. Once hemostasis was achieved, a two-layer closure was performed on the abdomen. An abdominal bandage was applied and the rats were allowed to recover from anesthesia before returning to the animal facility. We also inoculated ~5x10^6^ cells in Wistar rats (a progenitor of WAG/Rij rats) in two separate lobes of the liver via subcapsular injection in 12 rats after midline laparotomy was performed. The tumors were allowed to grow for 7 days and imaging was performed for 3 weeks. A single tumor underwent irreversible electroporation (IRE) with eight 100μs duration 1250 V/cm^2^-wave pulses separated by 100ms pulses) using ECM830 square-wave function generator (BTX, Harvard Apparatus, Holliston, MA).

### Ultrasound imaging

**Fig 2:**
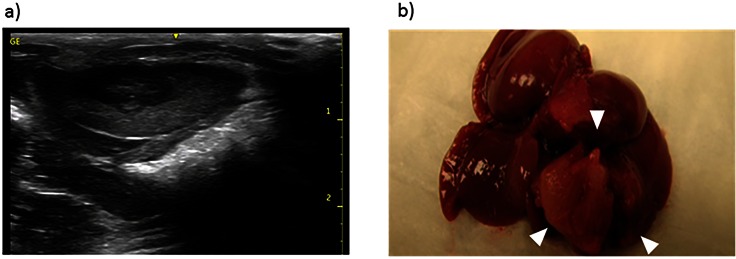
Ultrasound and Gross Pathology. (a) Transverse ultrasound images demonstrates a well-defined hypoehoic tumor in the anterior aspect of the left hepatic lobe. (b) Explanted gross specimen reveals tumors corresponding to the lesions seen on US (arrow heads).

Serial percutaneous ultrasound imaging (GE Venue 40, GE Healthcare, Waukesha, WI) of the liver and hepatic tumors was performed on two of the rats using a 12 MHz linear array probe (GE 12L-SC, GE Healthcare) at day 7, 14, and 21 post implantation (Fig 2A).

### Magnetic resonance imaging (MRI)

The rats were imaged on a 7.0 T MRI scanner (Bruker Clinscan Ettlingen, Germany) using a commercial four channel surface rat coil (Bruker) placed directly on the liver region. Animals were anesthetized using 2–3% isofluorane mixed with 100% O_2_ or medical grade air when performing the gas challenge experiment. Breathing artifacts on the images were avoided by implementing a triggered acquisition (when possible) using a MRI-compatible small animal respiratory gating system (SA Instruments, Stony Brook, NY). Body temperature was monitored continuously and controlled with a water-bed (SA Instruments). Prior to each imaging session, a 26 gauge tail vein catheter (Terumo Medical Corp, Somerset, NJ) was inserted and taped into place. A gadolinium based contrast agent (Magnevist, Beyer, Whippany, NJ) was used for the contrast enhanced study. During dynamic acquisition, contrast was injected at a concentration of 0.1 mM/kg. The following standard sequences were used for acquisition of the images: 1) a T2* weighted multi gradient echo (mGRE) TR = 300 msec, TE = 2,4, 8,12,16 msec, slice thickness 1 mm, in plane resolution 273 um; 2) a T1 weighted gradient echo sequence with TR = 500 msec TE = 3 msec which was repeated sequentially with a temporal resolution of 3.6 sec (120 repetitions) and the same geometry as 1; 3) a T2 weighted multi spin-echo sequence with TR = 1500 msec and TE = 30 msec also with same geometry as 1 and 2. For each animal and each time point, a set of anatomical images and parametric maps, which were generated using commercial medical image processing software JIM (Xinapse, West Bergholt, Essex, UK), were obtained. The T2* sequence was run twice, first with animal breathing 100% O_2_ and then with animal breathing medical grade air (5–10 minutes after switching O_2_ to room air). For the DCE data analysis, a two compartment pharmacokinetic model as well as semi-quantitative area under the curve approach was utilized. The arterial input function was obtained by drawing a region of interest around tracts of the iliac artery.

### Gross/Histopathology and Immunohistochemistry

**Fig 3:**
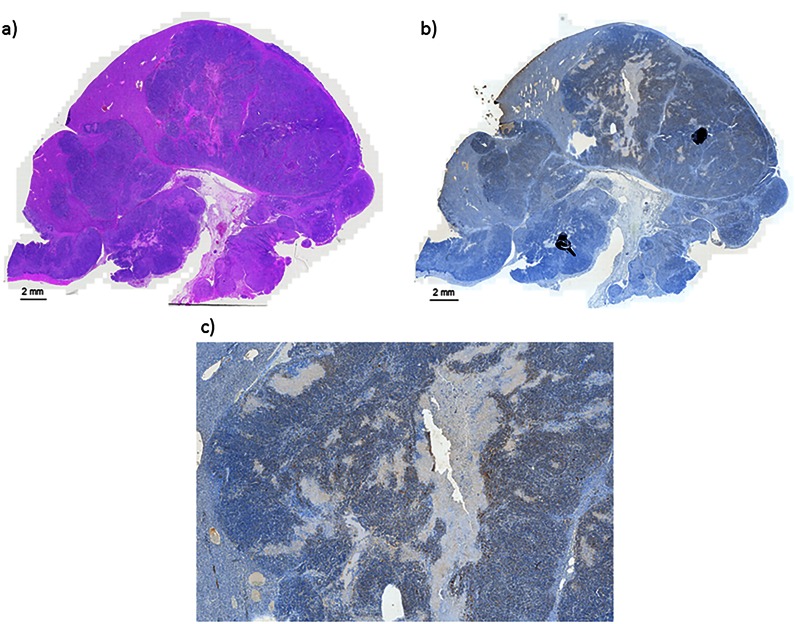
Histology and Immunohistochemisty. (a) H&E staining was performed confirming tumors within the liver. Microvessel density was evaluated with CD31 staining (b) 2.5x, (c) 20x. Scale bars represent 2 mm.

After final MR imaging, rats were euthanized with Euthasol (Virbac, Fort Worth, TX) and the livers were explanted. (Fig 2B). The formalin fixed tumors were then sectioned and sent for hematoxylin and eosin (H&E) staining to assess for tumor necrosis and microvascular invasion (Fig 3A). In addition, the tumor specimens underwent immunohistochemistry staining with CD31 to assess for microvessel density (Fig 3B and 3C). Each of the specimens were evaluated by assessing six randomly chosen 20x high power fields (hpf) in both the center and periphery of the tumor. The number of CD31 positive staining cells in each hpf were counted and the mean values were determined.

### Statistical analysis

A Student's t test was used to compare the CD31(+) cellular infiltration in the central and peripheral aspects of the tumor.

## Results

### Proliferation index

At 24 hours, the 6 well plate harbored 2 x 10^5^ cells, and there were 18 x 10^5^ cells in each of the three flasks. Therefore, the estimated doubling time of CC-531 cell line *in vitro* was 23 hours and 53 minutes.

In the animal model, 6/6 rats grew tumors with interval increase in size over the 21 day study period.

### Ultrasound

Ultrasound of the two tumor bearing rats demonstrates well-defined hypoechoic masses along the anterior aspect of the liver. (Fig 2A)

### MR imaging

**Fig 4:**
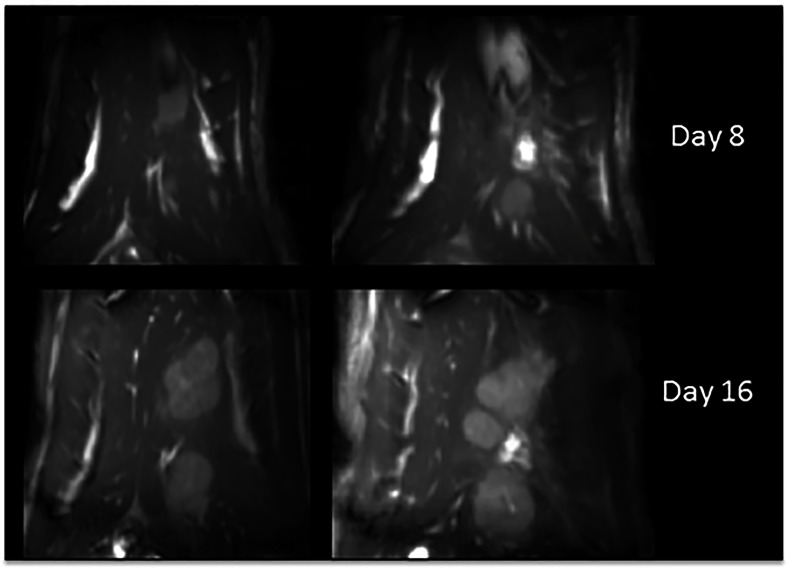
MR imaging. Coronal contrast enhanced MRI images for the same rat at 8 and 16 days post injection of tumor cells, demonstrating rapid increase in tumor volume.

**Fig 5:**
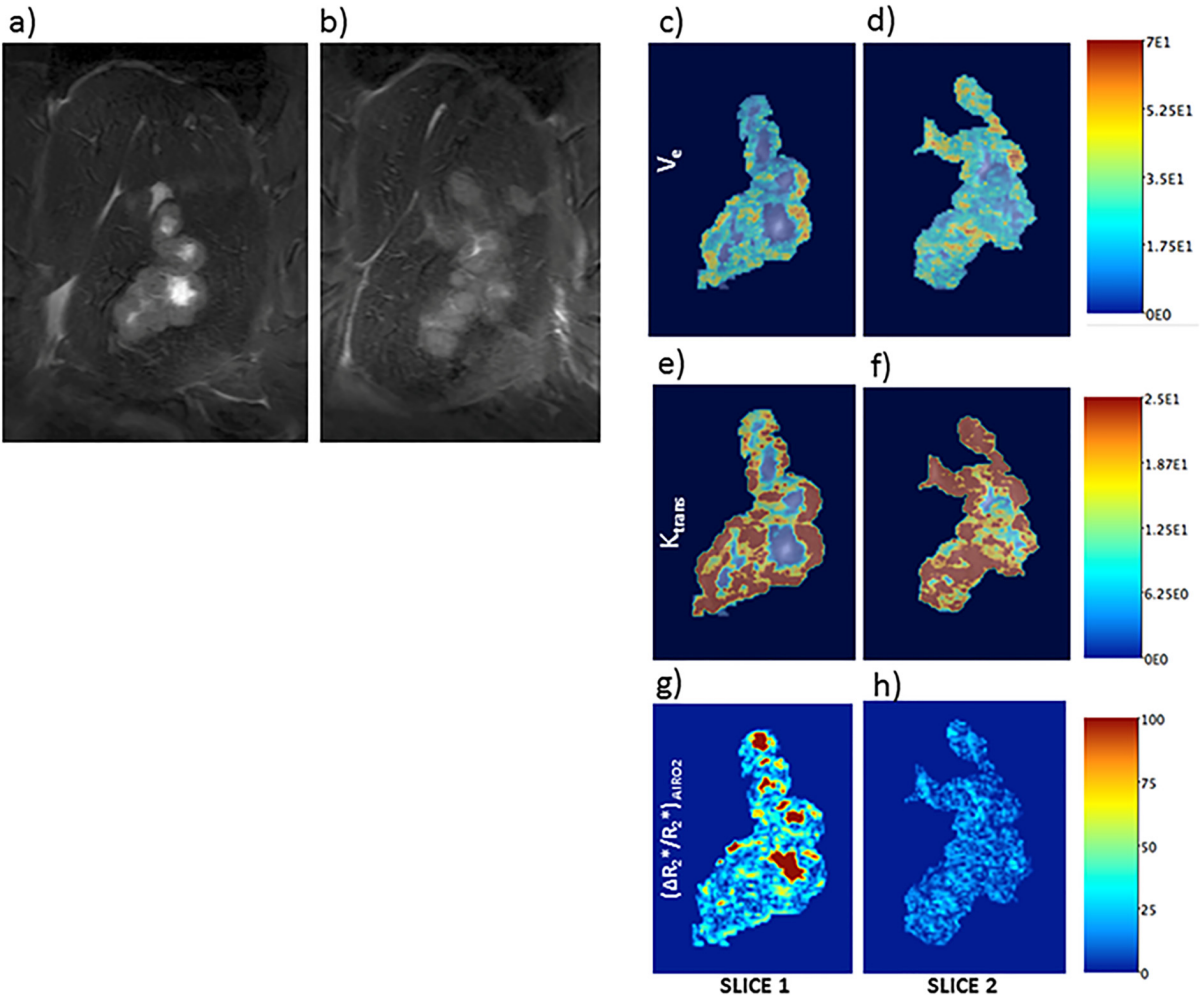
Quantitative MRI. Shown in (a) and (b) are two adjacent coronal slices of a representative rat liver with advanced stage tumor. Tumor lobes are identifiable as hyperintense relative to adjacent normal liver tissue within T2-weighted anatomic images. Shown In panels (c)–(h) are the corresponding parametric maps produced from dynamic contrast enhanced (DCE) image series. (f)These depict the vascular volume and permeability of tissues within the tumor region. R2* difference map from gas-challenge BOLD MR studies in this tumor model are shown in panel; significant heterogeneity in both gas-challenge signal changes and quantitative DCE measurements suggest a heterogeneous tumor microenvironment with regional variations in perfusion, tissue oxygenation and blood volume.

All T2 weighted MR images of the liver tumors exhibited hyperintensity compared to the normal liver parenchyma. The contrast between tumor and normal liver increased over time. The dynamic contrast enhanced (DCE) MR showed increased tumor enhancement (Fig 4) with different regions taking up contrast at different rates suggesting different degrees of vascularization. The BOLD/gas challenge data (DeltaR2* parametric images) also revealed regions with different quantitative values suggestive of a complex tumor microenrvironments characterized by different degrees of cellular hypoxia and microvasculature abnormalities (Fig 5). Both anatomic and quantitative MR images demonstrated heterogeneity within the tumor microenvironment. Wistar rats implanted with CC-531 cells showed bilobar tumor growth in 11 of 12 rats on day 7. However, 3 rats had metastatic implants along their peritoneum and the liver was unable to be successfully mobilized.

### Irreversible electroporation

**Fig 6:**
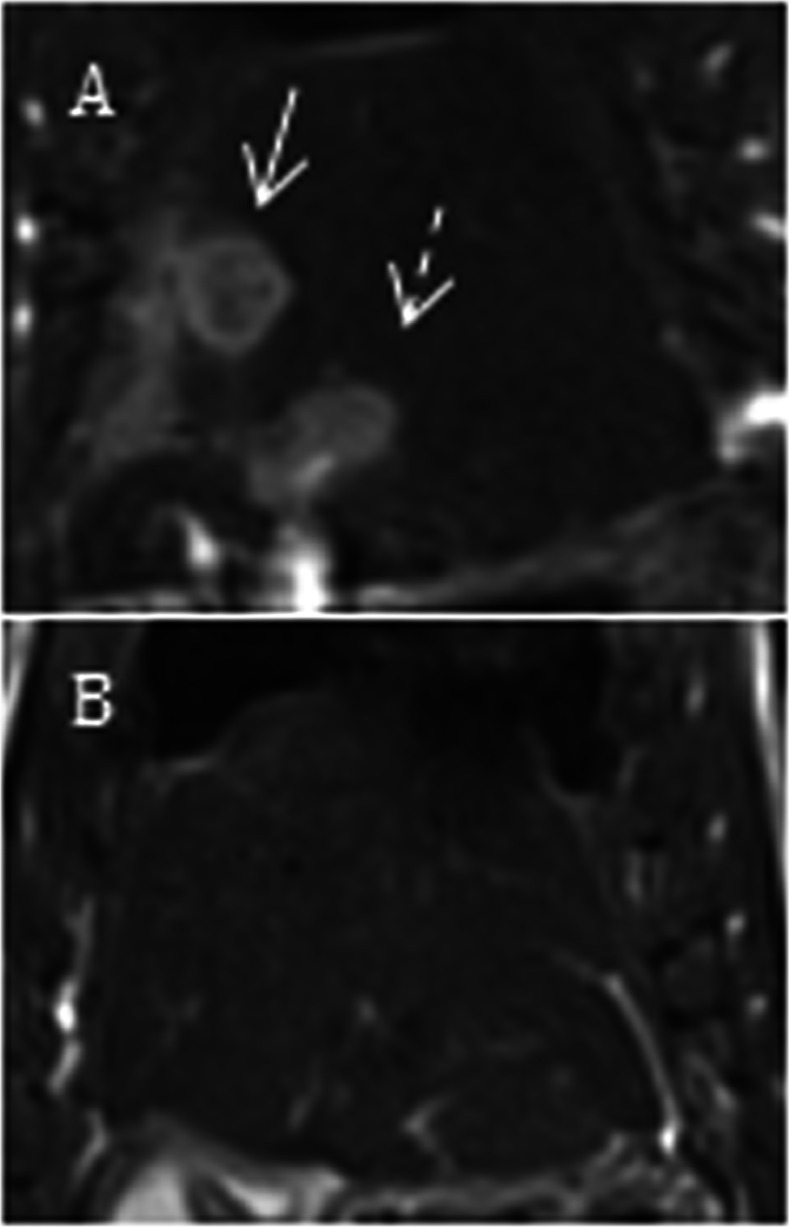
Irreversible Electroportation Therapy. Representative coronal T2 weighted MR images from two Wistar rats prior to IRE (A.I) and 7 days post IRE (A.II). White arrows = control tumors, and dashed arrows = tumors that under went IRE.

8 rats underwent irreversible electroporation of a single tumor. Post-IRE MR images after 7 days demonstrate regression of both tumors in Fig 6.

### Histology and Immunohistochemical analysis

H&E confirmed tumor growth in all rats (Fig 3A). The mean number of positively stained CD31 cells in the central aspect of the tumor was 101 (±32) (range 74–162) per 20x hpf versus 96 (±45) (range 44–160) in the tumor periphery. There was no statistically significant difference (*p* = 0.832).

## Discussion

Animal models assists in the generation of novel information about the metastatic phenotype, and provides a valuable, preclinical system for testing anti-metastatic therapies (reviewed in [17], [18]). Development of an in vivo model to study metastases is often challenging and largely dependent on immune compromised animals and cells of primary tumor origin [19], [20]. Spontaneous metastasis models are commonly initiated by the orthotopic injection of the cells reflecting the origin of the cancer [21] or implanting patient derived tumors in animals (reviewed in [22]). A large body of literature on experimental metastasis animal models points to intravenous or intracardiac injection of cancer cells [17]. Although spontaneous metastasis models offer real scenario of metastatic spread of disease, time required to establish metastatic lesions and poor success rates are often prohibitive for this approach. On the other hand, experimental metastasis models may not reach the major metastatic site of the particular cancer type. Recent focus has shifted to use immune competent animals and implantation of metastatic cell lines to recapitulate the real tumor microenvironment [23].

In interventional oncology, many animal models of disease have been utilized to understand ablative and trans-catheter intra-arterial therapies. To date, there are many models of orthotopic hepatocellular carcinoma in a rat model, including the Morris (McA-RH7777), [24] Novikoff (N1S1),[24, 25] and AS30D [26] and VX2 in the rabbit model. [27] However, as interventional oncologists expand their practice to treat liver dominant hepatic metastases, little is known about treatment efficacy. Translational models of metastatic disease allow for better understanding of safety and efficacy of the different treatment modalities. However, to be reliable and effective, the animal model must mimic clinical disease. In addition, this model must allow an easy way to detect changes following therapy and should provide insight into the functional and cellular changes, which occur as a result of an intervention and potentially provide a set of quantitative therapeutic biomarkers.

Developing this type of preclinical animal model of colorectal liver metastases can be challenging. One approach to achieving liver metastases is to implant colon cancer cells directly into the liver. [28] This model however does not truly represents a metastatic model, but rather represents heterotopically implanted cells in the liver. To account for this, colon cancer cells have been injected into the spleen, [29] or portal vein directly [30] and over time liver metastases develop. The liver tumors therefore represent metastatic disease; however, these hepatic tumors are infiltrative, limiting the ability of imaging to characterize or determine treatment efficacies. In addition, these animals develop distant metastases, hampering long term survival studies. Genetically altered animals are another approach to developing metastatic disease; however, these models again do not truly represent the vast majority of metastases, and therefore the results are not generalizable. An alternative approach is to expose animals to a carcinogen, or inject colon cancer cells orthotopically, [31] [32] and wait for the animal to develop the primary cancer with distant metastases. Though this represents a true metastatic model, it requires a considerable amount of time, and by the time metastatic disease develops, the animals are too ill to undergo therapy. Perhaps the most elegant approach, was performed by a group that treated WAG/Rij rats with 30 mg/kg of 1,2 dimethylhydrazine (DMH) subcutaneous injections for 6 weeks. [33] [34] Approximately, 40 weeks later the rats developed moderately differentiated colorectal adenocarcinoma, which then metastasized to the liver. The liver metastases were then harvested and the CC-531 cell line was generated. These cells can then be inoculated into the liver, creating a single focus of metastatic disease. Though this is not a “true” metastatic model, it serves as a metastatic model as metastatic cells are inoculated into the liver.

The current study focused on validating this model of colorectal liver metastases, which can be used rapidly and reliably in a preclinical setting. By using a subcapsular injection of metastatic cells, distinct tumors develop and progress in situ. Changes in their microenvironment can be monitored non invasively and continuously early after implantation [35]. Though this model was first described in 1985, to date and to the best of our knowledge this is the first attempt to fully characterize this tumor model using imaging data in combination with histological evaluation. [34] The present study demonstrates that detection of tumors is readily achieved using ultrasound early after implantation. Visualization with ultrasound enables percutaneous interventional treatment approaches. In addition, multiparametric MR was shown to effectively track changes in tumor microenvironment as it progresses naturally and following treatment. This tumor progression was tracked using a combination of clinically relevant imaging techniques (ultrasound, BOLD tumor imaging and DCE) with the dual purpose of characterizing and validating the CC-531 as a colorectal liver metastases model which mimics human disease and demonstrating how advanced multi-parametric imaging can help track changes associated with tumor progression and response to therapy. Specifically, this study demonstrates that it is possible to reliably use this animal model in combination with non-invasive and clinically applicable measurements of tumor size, cellular makeup, vascularity and hypoxia status to efficiently test safety and efficacy of new therapeutic approaches. Combined statistical analysis of this combination of MRI parameters could potentially predict response to therapy, while providing information on significance of cellular changes that modify the tumor microenvironment. Due to the limitation of syngeneic WAG/RijCmcr rats supply, we chose to show the proof of concept for tracking tumor growth using MR imaging, we used wistar rats as animal models and showed regression of tumor growth in T2 weighted images of IRE treated rats. However, our ongoing studies with interventional therapeutics in WAG/RijCmcr rat models warrants quantitative measurement of the tumor growth characteristics.

In conclusion, CC-531 is an immunocompetent and orthotopic translational rodent model of colorectal liver metastases mimicking tumor microenvironment with liver metastases easily established, tracked and characterized by US and MR imaging.
